# Premaxilla: up to which age it remains separated from the maxilla by a suture, how often it occurs in children and adults, and possible clinical and therapeutic implications: Study of 1,138 human skulls

**DOI:** 10.1590/2177-6709.23.6.016-029.oin

**Published:** 2018

**Authors:** Mariana Trevizan, Paulo Nelson, Solange de Oliveira Braga Franzolin, Alberto Consolaro

**Affiliations:** 1 Universidade de São Paulo, Faculdade de Odontologia de Ribeirão Preto, Programa de Pós-Graduação de Odontopediatria (Ribeirão Preto/SP, Brazil).; 2 Universidade de São Paulo, Faculdade de Odontologia de Ribeirão Preto, Departamento de Clínica Infantil (Ribeirão Preto/SP, Brazil).; 3 Universidade do Sagrado Coração, Departamento de Odontologia (Bauru/SP, Brazil).; 4 Universidade de São Paulo, Faculdade de Odontologia de Bauru (Bauru/SP, Brazil).

**Keywords:** Premaxilla, Maxillofacial development, Maxilla, Sutures.

## Abstract

**Objective:**

To evaluate topographic and temporal aspects of premaxillary bone and premaxillary-maxillary suture, since they are fundamental anatomical elements little explored clinically.

**Methods:**

1,138 human dry skulls were evaluated, of which 116 (10.19%) of the specimens were children, and 1,022 (89.81%) were adults. The skulls were photographed and the percentage of premaxillary-maxillary suture opening was determined. Subsequently the data were tabulated and submitted to statistical analysis, adopting a level of significance of 5%.

**Results:**

The progression of premaxillary suture closure from birth to 12 years of age was 3.72% per year. In 100% of the skulls up to 12 years, the premaxillary-maxillary suture open in the palatal region was observed, while 6.16% of adults presented different degrees of opening.

**Conclusions:**

The premaxilla exists in an independent way within the maxillary complex and the presence of the premaxilla-maxillary suture justifies the success of anteroposterior expansions to stimulate the growth of the middle third of the face, solving anatomical and functional problems.

## RESUMO

**Objetivo:**

avaliar aspectos topográficos e temporais do osso pré-maxilar e da sutura pré-maxilar/maxilar, por serem elementos anatômicos fundamentais pouco explorados clinicamente.

**Métodos:**

foram avaliados 1.138 crânios secos humanos, sendo 116 (10,19%) dos espécimes crianças e 1.022 (89,81%) adultos. Os crânios foram fotografados e determinou-se a porcentagem de abertura da sutura pré-maxilar/maxilar. Posteriormente, os dados foram tabulados e submetidos a análise estatística, adotando-se nível de significância de 5%.

**Resultados:**

a progressão de fechamento da sutura pré-maxilar/maxilar do nascimento aos 12 anos de idade foi de 3,72% ao ano. Em 100% dos crânios até 12 anos, observou-se a sutura pré-maxilar/maxilar aberta na região palatina, enquanto 6,16% dos adultos apresentavam diferentes graus.

**Conclusões:**

a pré-maxila existe de forma independente dentro do complexo maxilar e a presença da sutura pré-maxilar / maxilar justifica o sucesso de expansões anteroposteriores para estimular o crescimento do terço médio da face, solucionando problemas anatômicos e funcionais.

## INTRODUCTION

The premaxilla and the upper lip are formed between the fourth and seventh weeks of intrauterine life^1^ ^,^ ^2^. After that, the embryo’s head elevates and no longer touches the cardiac prominence.^3^ The mandible then grows, which creates room for the tongue to move down, at the same time that the palatine processes proliferate and elevate toward midline in a hinge movement,^4^ to join and form the secondary palate^1^ ^,^ ^2^ after leveling. 

Around the seventh month of intrauterine life, there is a change in blood supply to the face at a critical time for the development of the face and the palate. The premaxilla begins to ossify at this stage,^5^ and the center of ossification is separate from the actual maxilla.^6^ In the anterior region, it levels with the primary palate, preserving the incisive foramen and canal in the midline, which are derived from the primary and secondary palates and contain vessels, nerves, glands and segments or remnants of the nasopalatine duct.^7^

In the first years of postnatal life, cranial growth predominates over facial growth.^8^ ^,^ ^9^ During this period, mandibular growth is exuberant, while the growth of the maxillary complex is limited. The maxillary complex grows toward the anterior and inferior region^10^ in a predominantly horizontal movement in the first decade of life and a vertical movement in the second decade. The morphological and clinical therapeutic descriptions of the maxillary complex hardly ever mention the anterior segment,^11^ although it is an independent bone that only later overlaps into a semi-independent bone. 

The premaxilla, where the four maxillary incisors are,^12^ ^,^ ^13^ develops from the primary palate^5^ ^,^ ^14^ and is closely related to the development of the human face.^12^ The limits of the premaxilla are defined by a suture that goes from the incisive foramen to the region between the lateral incisors and canines, with a variable position between these teeth.^15^ This gives shape to what has formerly been called the incisive bone.^5^ This suture goes down from the junction of the maxillary and premaxillary growth centers, close to the lower portion of the pyriform aperture, to the alveolar margin in the region of the canine, crossing the palate to the incisive foramen.^16^

Four parts of the premaxilla may be identified: 1) its body, which is continuous with the maxilla; 2) its alveolar portion, which holds the teeth; 3) the palatine process; and 4) the stenonianus (infravomerine) process, which fuses with the cartilage of the nasal septum and vomer.^6^

The anatomy of the premaxillary area has not been fully described^17^. The time in its growth and development when the suture between the premaxilla and the maxilla fuses, so that they become a single bone, has not been determined.^18^ Abnormal growth in this region may be correlated with malformations, such as prognathism, deep bite and protrusion.^12^

Comprehension of the mechanism of formation and the causes of orofacial developmental disorders requires full knowledge of embryology and anatomy.^7^ The understanding of premaxillary development and how it is associated with the maxilla may: 

### 1) Help to understand the etiology of cleft lip and palate and its subsequent effects on craniofacial growth, so that more refined and pertinent treatments of these developmental disorders may be planned

Initial facial development is not associated in any way with the ossification centers, which form later than the embryonic processes that give origin to facial tissues and components. Bone sutures are not the places where the embryonic processes touched each other. These are two independent phenomena, especially in relation to the time when they occur.^7^

Cleft position does not always coincide with the premaxillary-maxillary suture,^12^ ^,^ ^15^ ^,^ ^19^ because bone development does not match primary facial development. Face formation lines are not identical to those where bone growth centers meet in all sutures, including the premaxillary-maxillary suture. Bilateral cleft lip and palate produce a premaxillary protrusion, which includes the soft tissue below the nose and the teeth in these region.^20^ Treatment follows several stages, such as alveolar bone grafting, with a graft that may be autogenous or synthetically manufactured^22^ and is used to fix the cleft. The existence of a premaxilla as an independent bone makes it possible to move it^18^ to reduce the cleft before grafting and to place it at a more favorable position.

### 2) Establish the principles and promote the development of new treatments for the changes in development and growth of the maxillary complex and the midface using the anteroposterior expansion of the maxilla

Orthodontic appliances may lead the premaxilla to a more beneficial anterior position^23^ ^,^ ^14^ by stimulating the maxillary sutures.^23^ The stimulation of the premaxillary-maxillary suture results in the development of this region^18^ ^,^ ^23^ ^-^ ^25^ by means of inflammation and repair that culminates in remodeling the maxillary complex,^26^ which may be used for the non-surgical protraction of the maxillary complex following, for example, the Ertty Gap III^®^ protocol.^27^ Therefore, the suture between the premaxilla and the maxilla may be the adequate biomechanical point for interventions in cases of Class III malocclusion, in which the diagnosis indicates insufficient maxillary complex development. The existence of a premaxilla as an independent bone allows for sutural movement and periosteal bone growth to correct certain malocclusions, with a reduction of risk and severity and even eliminating the need for surgery.

### 3) Establish and promote existing and new treatments for cases of nasal obstruction of newborns due to congenital pyriform aperture stenosis

Nasal obstruction in infants is a potentially serious condition^28^, as it may lead to respiratory failure of the newborn^29^. One of its causes is congenital pyriform aperture stenosis, with a narrowing of the anterior third of the nasal fossa caused by excessive growth of the medial nasal process of the maxillary complex.^30^ The intermaxillary bone, or premaxilla, is the main limit of the pyriform apertures, and parts of this bone may be occasionally seen lateral to the pyriform apertures up to about the fifth year of life, together with the nasal bone, which closes the pyriform aperture and touches the frontal bone.^12^ The existence of the premaxilla as an independent bone makes it possible to move it^18^ and thus remove airway obstructions.

Knowledge of all the aspects of the premaxillary bone and premaxillary-maxillary suture is essential and fundamental for clinical and therapeutic uses. Therefore, the objectives of this study were:

» To determine the frequency of an open premaxillary-maxillary suture in dry human skulls of children and adults.
» To analyze the topography of the premaxillary-maxillary suture to understand its function and relevance in the craniomandibular skeleton.
» To estimate the time of premaxillary-maxillary suture closure in human development.
» To evaluate the implications of these results for treatment options for situations, conditions and diseases that affect this anatomic region.

## MATERIAL AND METHODS

### Ethical issues

This project was submitted to and approved by the Ethics in Research Committee (ERC) of the *Faculdade de Odontologia de Ribeirão Preto, Universidade de São Paulo* (FORP-USP, under code CAAE 61308316.0.0000.5419), as well as by the ERC of the *Faculdade de Odontologia de Piracicaba, Universidade de Campinas* (FOP-UNICAMP, under code CAAE 61308316.0.3002.5418), and the ERC of the *Escola Paulista de Medicina, Universidade Federal do Estado de São Paulo* (EPM-UNIFESP, under code CAAE 61308316.0.3003.5505). This study used skulls of the Discipline of Anatomy of the *Faculdade de Odontologia de Bauru, Universidade de São Paulo* under permission from the professor responsible for its anatomic collection. Informed consent for the use of skulls was waived, but there was explicit authorization from the people responsible for the use of these specimens in each institution participating in the study.

### Sample

Skulls were included in the study regardless of sex, ethnicity or age. Exclusion criteria were the impossibility to examine the site of the premaxillary-maxillary suture visually, cranial deformities and skulls of individuals that had syndromes.

### Determination of age

Age was determined by evaluating the approximate phase of deciduous and permanent tooth eruption, as described by Schour and Massler (1941), and confirmed using the method described by Rai et al^31^(2014). Age groups were classified into scores, as described in Table 1. The determination of approximate age was not performed for adult skulls.

**Table 1:** Scores for approximate age.

Score	Age group
0	Up to birth
1	0 to 3 years
2	3 to 6 years
3	6 to 9 years
4	9 to 12 years
-	Older than 12 years

### Determination of premaxillary-maxillary suture presence

**Figure 1:**
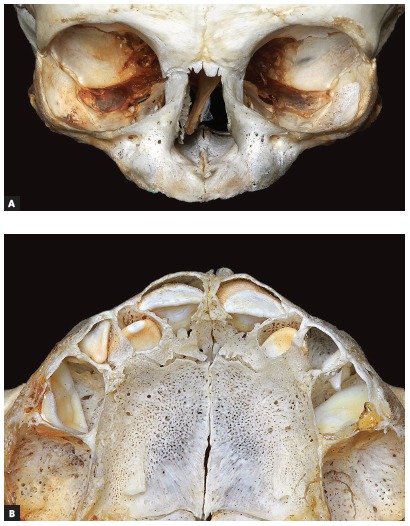
A) Facial region; B) palatal region. Note the greater width of the premaxillary-maxillary suture, and its linear shape, perpendicular to the midpalatal suture.

**Figure 2:**
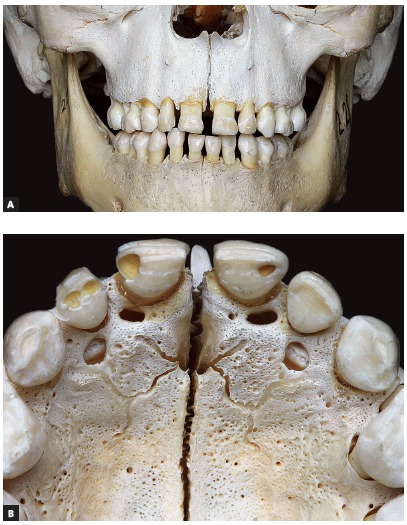
A) Facial region; B) palatal region. Exuberant and serpiginous aspect of premaxillary-maxillary suture.

**Figure 3:**
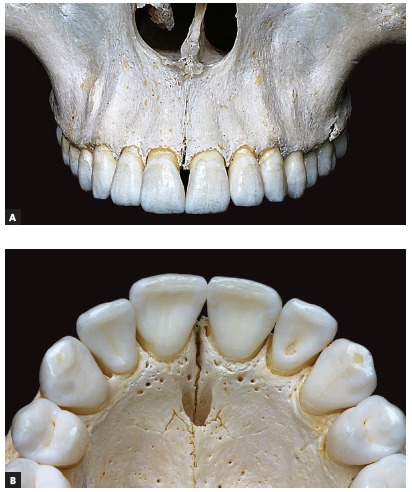
A) Facial region; B) palatal region. Simple and linear aspect of premaxillary-maxillary suture.

**Figure 4:**
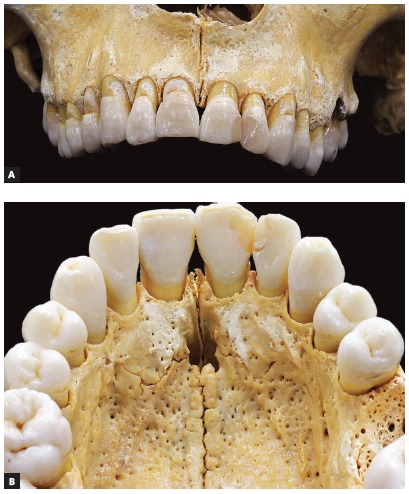
A) Facial region; B) palatal region. Smaller width and origin in lateral wall of incisive foramen.

All skulls were visually examined by two observers. The analyses were conducted following the different topographic regions on buccal and palatal views (Figs 1, 2, 3 and 4) because this bone does not develop uniformly or symmetrically in all regions. All skulls of individuals under 12 years of age were photographed. The skulls of older individuals were photographed only in cases of previous visual confirmation of the existence of the premaxillary-maxillary suture.

### Determination of the opening/closing ratio of the premaxillary-maxillary suture

**Figure 5:**
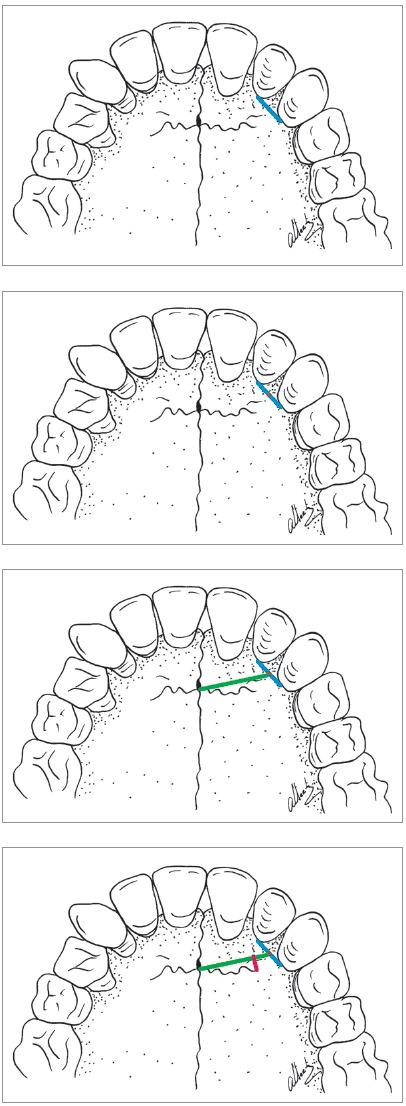
Sequence to determine the percentage of premaxillary-maxillary suture opening/closure.

To determine the percentage of opening of the premaxillary-maxillary suture, two observers independently identified the side on which the suture had the longest opening and traced a straight line from the incisive foramen to the middle point between the maxillary lateral incisor and the maxillary canine in the palatal region. The end of the suture was projected orthographically onto this straight line, and the percentage of opening was calculated according to the ratio between the length of the open segment projection and the total segment length. The evaluation of suture opening percentage considered that 100% open were those sutures that reached the end of the straight line, and 0%, those that did not extend from the incisive foramen, as shown in Figure 5.

### Statistical analysis

**Figure 6:**
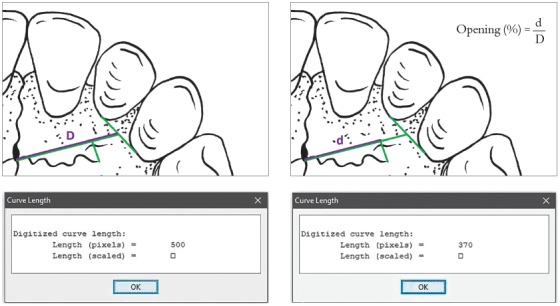
Sequence to measure premaxillary-maxillary suture opening in pixels.

The data collected were tabulated using Microsoft Excel 2013 software. The segments described in the last section were measured using the software Plot Digitizer v. 2.6.8, which provides distances between two selected points in pixels (Fig 6). After that, data were analyzed using PAST software (Paleontological Statistics Software Package for Education and Data Analysis, National University of Ireland, Galway, Ireland). The Pearson correlation test was used to analyze the percentage of premaxillary-maxillary suture opening and the number of deciduous and permanent teeth, age and age scores. The level of significance was set at 5%.

## RESULTS

**Figure 7:**
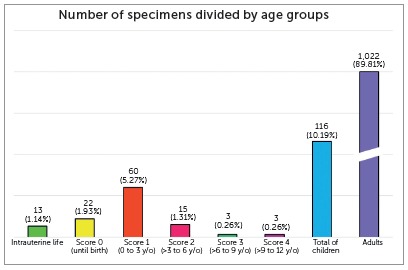
Number of specimens divided by age groups.

Of the 1,138 specimens evaluated, 116 (10.19%) skulls were of infants and children, and 1,022 (89.81%), of adults. Of the infant and child skulls, 13 were of individuals in intrauterine life and 103, extra uterine life, as shown in Table 2 and illustrated in Figure 7.

**Table 2:** Number of specimens according to age groups.

Age group	Number of specimens	% of specimens
Intrauterine life	13	1.14%
Score 0	22	1.93%
Score 1	60	5.27%
Score 2	15	1.31%
Score 3	3	0.26%
Score 4	3	0.26%
Total no. of children	116	10.19%
Adults	1,022	89.81%
Total	1,138	100%

The 13 skulls in the intrauterine life group had a gestational age of four to five months. They all had 100% opening of the premaxillary-maxillary suture on the palatal view, a finding that was repeated for all the 22 skulls of stillborns. An open suture was not identified on the frontal view of any of these specimens.

The 81 skulls of the extra uterine and not stillborn group belonged to children 6 months to 12 years old. The greatest frequency, 60 skulls, had score 1, that is, zero to three years of age; and 54% of them had 100% opening of the premaxillary-maxillary suture; the other 6 had openings ranging from 34.35% to 86.4%, with a standard deviation of 23.10% (Fig 12).

Table 3 shows skull distribution, mean premaxillary-maxillary suture opening percentage and mean number of deciduous and permanent teeth according to age scores. Closure percentage was recorded as the difference from the opening percentage (100% - opening%).

**Table 3:** Distribution of skulls of children according to mean premaxillary-maxillary suture opening and closure, and mean number of deciduous and permanent teeth according to age scores.

Age score	Number of skulls	Mean opening (%)	Mean closure (%)	Mean number of deciduous teeth	Mean number of permanent teeth
0	22	100	0	0	0
1	60	96.09	3.91	6.07	0
2	15	83.59	16.41	10	0.93
3	3	65.04	34.96	7.3	4.67
4	3	56.04	43.96	0.67	11.33
Total	103				

**Figure 8:**
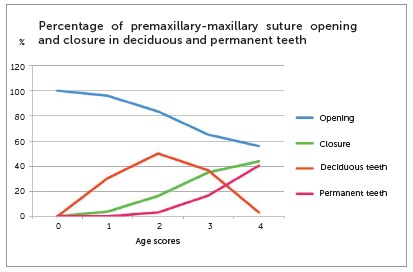
Mean percentage of premaxillary-maxillary suture opening, mean percentage of deciduous teeth and mean percentage of permanent teeth according to age score.

Figure 8 replicates the data in Table 3, but shows them as percentage of deciduous teeth, considering 20 teeth as complete deciduous dentition (100%), and percentage of permanent teeth, considering 28 teeth (100%) at age 12 as complete permanent dentition. The correlation between percentage of permanent teeth and premaxillary-maxillary suture was negative for opening and positive for closure. 

The Pearson correlation test was used to quantify the correlation of premaxillary-maxillary suture closure and number of permanent teeth, number of deciduous teeth, age and age score. Results and p values are shown in Table 4. 

**Table 4:** Pearson correlation between premaxillary-maxillary suture closure percentage and number of deciduous and permanent teeth, age and age scores.

	Closure of premaxillary-maxillary suture (%)	p value
No. permanent teeth	r= 0.5294	8.95E-09
No. deciduous teeth	r= 0.2907	0.0028976
Age	r= 0.6142	5.18E-12
Age score	r= 0.5550	1.17E-09

**Figure 9:**
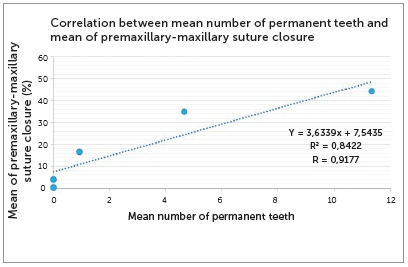
Correlation between percentage of premaxillary-maxillary suture closure and mean number of permanent teeth.

The highest correlation coefficient was that between the mean number of permanent teeth and the percentage of premaxillary-maxillary suture closure (r = 0.9177), at a test power of α=1.0 (Fig 9).

**Figure 10:**
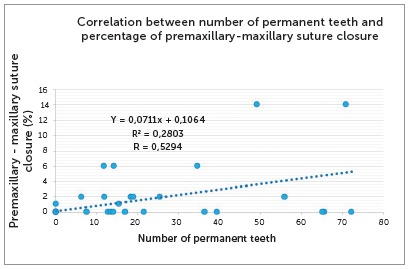
Correlation between percentage of premaxillary-maxillary suture closure and number of permanent teeth.

**Figure 11:**
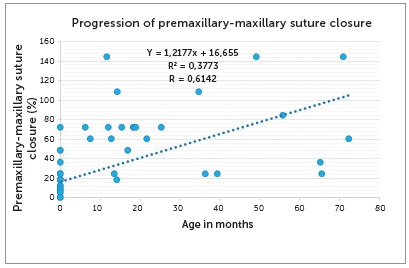
Correlation between age in months and percentage of premaxillary-maxillary suture closure: progression of premaxillary-maxillary suture closure.

The analysis of number of permanent teeth revealed a value of r=0.5294 (p= 8.95E-09), which indicates a moderate positive correlation. The linear regression equation was y=0.0711x+0.1064. Figure 10 shows the regression line, the line equation and the values of r^2^ and r. Figure 11 gives an example of the association between age in months and percentage of premaxillary-maxillary suture closure. 

According to data for the skulls in the zero-to-12 years group and using the line equation and age in years, we found a projection of premaxillary-maxillary suture closure of 3.72% per year.

Of the 1,022 adult skulls, 959 (93.84%) had a completely closed suture, that is, there was no suture, whereas 63 (6.16%) had different percentages of open and partially open sutures (Figs 13 and 15).

**Figure 12:**
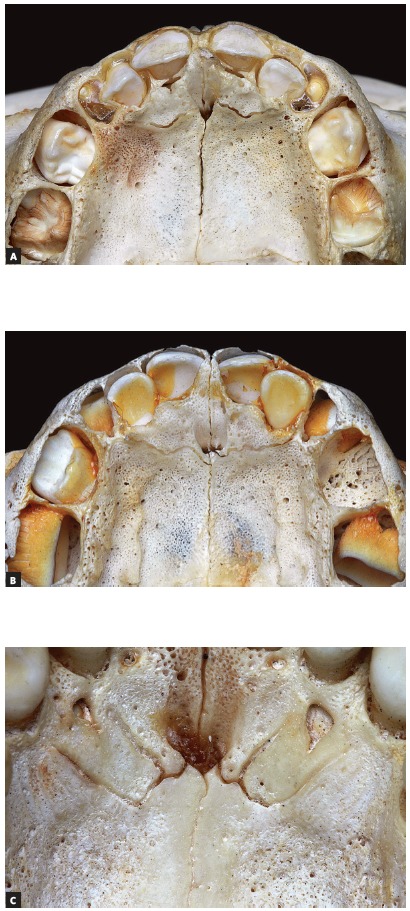
Morphological patterns of premaxillary-maxillary suture in children, with irregular shapes that are simple or complex, but always greatly variable.

**Figure 13:**
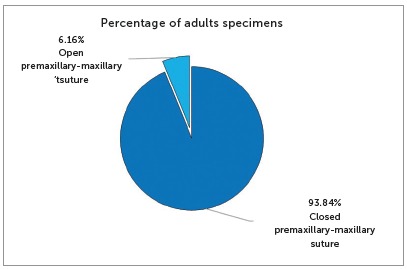
Prevalence of adult skulls with open premaxillary-maxillary suture and closed premaxillary-maxillary suture.

**Figure 14:**
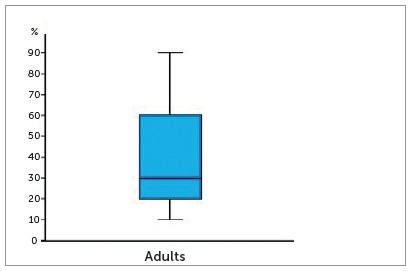
Boxplot of the percentage of open premaxillary-maxillary sutures in adult skulls.

**Figure 15:**
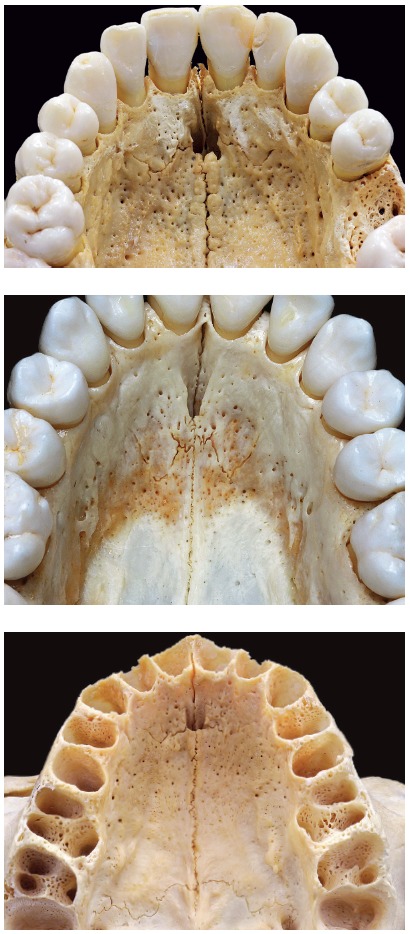
Morphological patterns of premaxillary-maxillary suture in adults: note great morphological variability.

Mean percentage of premaxillary-maxillary suture opening in the 63 skulls with a opening suture was 41.11%, with a standard deviation of 25.28%, a minimal value of 10%, a maximal value of 90%, and a median value of 30%. Figure 14 shows a boxplot with the minimal and maximal values, median value and quartiles.

## DISCUSSION

Neglected and still denied today by many scholars, the existence of the premaxilla offers treatment options to promote the growth of the mid third of the face, which may be a solution for some severe anatomical and functional problems.

In several areas of human development, no other element has raised as much controversy and discussion as the premaxilla. However, these facts do not justify its absence or little importance in innumerable Anatomy, Orthodontics and Odontopediatrics textbooks, among others,^13^ ^,^ ^16^ ^,^ ^32^ ^,^ ^33^ especially when its relevance is taken into consideration.^6^ ^,^ ^18^ Classified as a transient osseous element, with certain proper ossification centers, and subsequently unified to the maxilla,^6^ ^,^ ^13^ ^,^ ^18^ ^,^ ^34^ ^-^ ^36^ denying its existence is a basic conceptual mistake.^33^ ^,^ ^38^ ^-^ ^41^

The resistance to its recognition probably results from its early closure in the facial region,^16^ ^,^ ^42^ ^-^ ^44^ that is, in the alveolar part with facial process, which supposedly occurs in the first trimester of prenatal life.^16^ The earliest age found in this study was for a specimen of 16 weeks of intrauterine life, in which no opening of the suture was seen on the frontal view. Therefore, its closure in this region occurred at four months of intrauterine life.

The absence of a suture on the facial side results from the fusion of the nasal and incisive bone processes of the maxilla, which, however, remains patent in the palatal region during all early childhood.^13^ ^,^ ^45^ Despite that, it may sometimes persist into adulthood, as demonstrated in this study. This is an indication of the development of the human face, as the human premaxilla is similar to that of other mammals in shape, sutural limits, ossification, place and function, except for its absence on the facial side in certain stages of development^6^. In the newborn skull, sutures are widely open to allow for bone growth. Before they grow, a large number of bones, over 300, are independent, but that number goes down to 206 in adults because of the unification of several bones.^46^

In 2015, Botti et al^47^ reported that premaxillae are difficult to identify in studies of human anatomy, but argued that denying the existence of premaxillary bones would be incompatible with the successive development of the primary and secondary palates, as well as with the persistence of the incisive canal as a vestige of its articulation to form the hard palate.

The agenesis of the premaxillary bone in some diseases^48^ ^,^ ^49^ makes its existence and autonomy obvious, because the bone posterior to the premaxillary-maxillary suture, the maxillary bone, is present and functional.

Variability in suture obliteration time may be one more of the reasons that make the premaxilla a singular bone that is so rarely studied or used for treatments. These findings have been confirmed in this study by the innumerable time differences in suture obliteration, which suggest that premaxillary-maxillary suture closure in the palatal region occurs as age advances. This process is similar to that of formation and growing of permanent teeth, which are also associated with age, as reported previously.^50^ The analysis of Figure 8 reveals a similarity between the curves of increase in the number of permanent teeth and increase in closure of the suture under study.

The morphological disappearance of the premaxillary-maxillary suture, at an earlier age or in adults, may be assigned to the vertical growth of the maxillary complex and, in consequence, of all hard palate. The growth of the vertical maxillary complex has been described in Embryology textbooks that report growth as craniomandibular^33^. This vertical growth makes palatal bone remodeling and reshaping more dynamic and constant, so that it responds to new functional and anatomical demands of craniomandibular growth. As this growth is constant and moves the hard palate down and forward, inevitably the premaxillary-maxillary suture tends to disappear, because is no longer submitted to functional demands after a certain period. 

Sex and nutritional status affect the time of appearance of both the ossification centers in an individual and, consequently, bone development,^51^ ^-^ ^53^ which may justify the large variation in the times of premaxillary-maxillary suture closure found in the present study. However, on the palatal side, this suture often remains patent during all early childhood. We found 100% opening of the premaxillary-maxillary suture in 54 (52.42%) specimens aged zero to three years, and three (20%) of those aged three to six years. No specimens aged more than six years had 100% opening.

The present results suggest that the premaxillary-maxillary suture closes earlier on the facial side, but remains open at different rates in the palatal region during childhood and, sometimes, into adulthood. This opening may be a biomechanical point for orthodontic and orthopedic action that may lead to favorable results both esthetically and anatomically.

At 12 years of age, the premaxillary-maxillary suture may remain open, as three (100%) skulls in that age group had an open suture in this study. The age group of specimens of older individuals could not be determined at a minimally accurate level.

According to the approximate estimation of premaxillary-maxillary suture closure from birth to 12 years defined in this study, closure occurs at a rate of 3.72% per year. Therefore, the earlier the intervention, the easier and more flexible is bone movement, and the greater are the chances of success. 

A suture is the connection between two bones, a narrow band of dense fibrous connective tissue that forms an immovable joint.^26^ ^,^ ^54^ Sutures unite bones and play an important role in growth, as they are remodeled by stimulation,^26^ ^,^ ^54^ which is the case of the sutures in the maxillary complex,^23^ including the premaxillary-maxillary suture.

The maxillary complex is composed of membranous, highly malleable bones whose sutures act as growth sites^12^ when stimulated to proliferate,^55^ ^,^ ^56^ that is, when the bones that are joined by these sutures are submitted to traction. The premaxillary-maxillary suture, called incisive suture in the past, outlines the palatine process of the premaxilla and the palatine process of the maxilla,^6^ and, therefore, generates growth in these areas when stimulated. Therefore, the manipulation of the premaxillary-maxillary suture may correct deficiencies in the horizontal development of the maxillary complex before bony bridges are created, in which case surgical expansion is necessary. This is the case of median palatal expansion, in which failure is associated with the skeletal maturation of the patient.^23^ ^,^ ^56^ ^-^ ^58^ In 1982, Schwartz^56^ found an association of success and failure of orthopedic protraction of the maxillary complex with premaxillary-maxillary suture closure.

The use of the Ertty Gap III^®^ protocol had excellent results in maxillary complex protraction in children up to 13 years of age. It is simple, has a low cost, and its esthetic results are satisfactory when compared with other appliances for the same purpose. With an intraoral system, there is greater collaboration by the patient, and the continuity of force application promotes better results than intermittent forces, which is usually the case of other appliances.^27^

In relation to the maxillary complex, which is divided into anterior, middle and posterior, the premaxillary bone is usually included in the maxilla. However, the present authors suggest that the term maxilla should refer only to the portion derived from the embryonic maxilla,^6^ ^,^ ^13^ and should not include the premaxillary bone, because the anterior, premaxillary segment is autonomous during a certain time of human development and extremely valuable anatomically and functionally. Moreover, the term premaxilla should remain in use for human beings, even after the incomplete or complete closure of the premaxillary-maxillary suture.^13^

**Figure 16:**
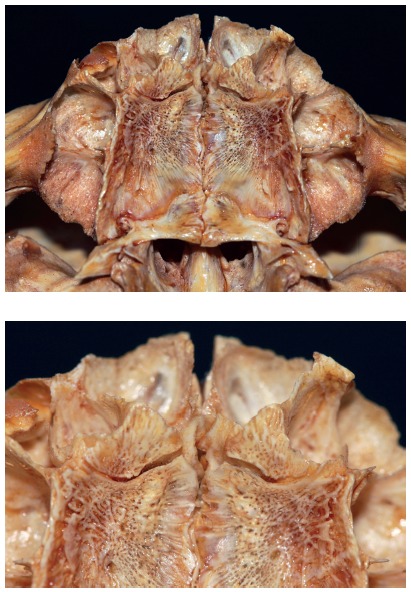
Early development of premaxillary-maxillary suture, about six months postnatally. Distribution of trabecular bone associated with sutures and cortical bone, probably due to growth vectors.

In adults, some remnants of the suture in the palatal region may be seen, which is in agreement with other findings.^12^ ^,^ ^43^ ^,^ ^50^ In Germany, Kadanoff et al^59^ found the premaxillary-maxillary suture or its remnants in 11.1% of the adults. In our study, in Brazil, we found it in 6.16% of the specimens, but it was not possible to accurately determine the geographic origin of each specimen. The premaxilla tends to disappear in adulthood.^12^ Therefore, expansion attempts to correct problems associated with positioning should be approached in childhood, and chances of success are greater at earlier ages because of bone development and maturation (Fig 16).

The association between premaxilla and maxilla has also been the focus of discussions and disagreement and is hard to understand.^32^ Some authors argue in favor of a “fusion theory”, in which there is the unification of the two bones,^16^ ^,^ ^60^ ^-^ ^62^ whereas others defend a theory of “excessive growth”, in which the premaxilla would be “embraced” by the maxilla.^13^ ^,^ ^42^ ^,^ ^63^ Shepherd and McCarthy,^6^ in 1955, however, demonstrated that the concepts above are not sufficient to describe the correlation between bones, and that what in fact occurs is resorption and replacement: while the premaxilla undergoes resorption, the maxillary trabecular bone fuses into the premaxillary bone. These three concepts are complementary and progressively explain the association between bones from the beginning to the end of the process. The growth of the maxillary complex generates several pressure and tension forces that direct its enlargement and growth and determine its final bone shape: these forces are called growth vectors. The explanation that we have for this anatomical continuity between the premaxilla and the maxilla claims that it occurs due to accelerated bone remodeling and reshaping in the palatal region to respond to vertical growth of the face, as previously mentioned. Bone remodeling and bone reshaping in the entire skeleton respond to functional demands, and their continuity eventually eliminates the premaxillary-maxillary suture when the functional demands for the premaxilla no longer exist. Therefore, the bone structures of both parts unite, and the suture tends to disappear without any perceptible sign in most human beings. Its most clarifying evaluation should be conducted using imaging studies. Occasionally it appears on occlusal radiographs of patients in the first decade of life, but it is thin and narrow, which makes its visualization difficult on conventional imaging studies.^23^ The attempt to visualize the suture on both conventional and digital occlusal radiographs of randomly selected skulls was not successful, as it was not possible to identify it in pilot studies carried out before this study. The use of CT may enable its visualization and measurement, but the efficiency and pertinence of tests and protocols should be evaluated before they are used. The use of CT scans in pediatric patients should be carefully evaluated and considered because of the latency of side effects for individuals with a longer life expectancy, such as children, who form a group at a greater risk than adults.

This study confirmed once more the existence of the premaxillary-maxillary suture and its frequency, and this confirmation supports the use of treatment strategies for the anteroposterior expansion of the maxillary complex.^27^ However, as mentioned before, this depends on skeletal maturity, and consultations and diagnoses at an early age are essential because treatment success is inversely proportional to development.

The simplicity of the fact that the premaxillary-maxillary suture explains a difficult concept, the anteroposterior expansion of the maxillary complex, may excite the sensitive nature of researchers. There is no better theory to explain the clinical results obtained.^55^ The movement of the premaxilla offers new opportunities for Orthopedics, Orthodontics and surgeries of this anatomical region to treat craniomandibular development and growth disorders.^18^ The resolution of conditions, such as the reduction of cleft lip and palate, the removal of nasal obstruction in congenital stenosis and correction of prognathism due to deficient development of the maxillary complex (Ertty Gap III^27^), involves several areas of knowledge in Dentistry and, therefore, their associations and prognoses are complex. Moreover, several correlations should be defined in future studies: 

1. Identify, limit and evaluate premaxillary-maxillary suture closure using imaging studies, including 3D reconstructions, as planning and prognosis parameters of orthodontic and orthopedic maxillary treatments. 

2. Correlate premaxillary-maxillary suture closure and midpalatal suture closure^65^. Late closure with opening of the midpalatal suture may be associated with the same phenomenon for the premaxillary-maxillary suture.

3. Investigate the association between premaxillary-maxillary suture closure or its prolonged opening with facial patterns or occlusion Classes I, II or III.^50^ Early or late closure of the premaxillary-maxillary suture may affect facial type.

4. Study the interference of midpalatal expansions using different appliances, such as Haas, Hyrax and MARPE, in the premaxillary-maxillary suture, as the anterior screws of the MARPE appliance are placed at the level of the third palatine rugae, very close to the premaxillary-maxillary suture. The effect that it may have on maxillary process movement should be studied, because these screws may not be located in the maxilla in all cases - and if the premaxilla with a suture is still present, results may be different.^11^ ^,^ ^66^

5. Study premaxillary-maxillary suture opening or closure and the intensification of childhood oral habits and how they may be associated. 

The existence of the premaxilla was used as a reference point at the time of Goethe to distinguish human beings from other mammals,^46^ as unusual as it may seem to be today. At that time, the premaxilla was seen as one of the characteristics that differentiated animals from human beings. When Goethe detected and described the human premaxilla to the scientific community, a taboo was broken and, at the same time, placed him in history as one of the precursors of Darwin’s theory of evolution. However, still today we find texts that deny the independent existence and identity of this anatomical and functional structure. The present results in a study of 1,138 dry human skulls allow us to argue that the premaxilla does exist!

## CONCLUSIONS

The results of this study suggest that:

1. The progression rate of premaxillary-maxillary suture closure from birth to 12 years of age was 3.72% per year.
2. All pediatric skulls up to 12 years of age had an open premaxillary-maxillary suture in the palatal region, at different opening percentages.
3. Adults may have an open premaxillary-maxillary suture.
4. The percentage of adults with an open premaxillary-maxillary suture was 6.16%.
5. The presence of a premaxillary-maxillary suture explains the success of anteroposterior expansion of the maxillary complex.
6. The existence of the premaxillary-maxillary suture supports the use of treatments for the growth of the middle third of the face to solve anatomical and functional problems.

## References

[1] Lubit EC (1978). Fissura labial palatina. Quintessência.

[2] Pashley NR, Krause CJ (1981). Cleft lip, cleft palate, and other fusion disorders. Otolaryngol Clin North Am.

[3] Ferguson MW (1981). Developmental mechanisms in normal and abnormal palate formation with particular reference to the aetiology, pathogenesis and prevention of cleft palate. Br J Orthod.

[4] Tuchmann DH, Haegel PH, Solère M (1969). Embriología cuadernos prácticos.

[5] Sperber GH (1976). Craniofacial embryology.

[6] Shepherd WM, Mc Carthy M (1955). Observations on the appearance and ossification of the premaxilla and maxilla in the human embryo. Anat Rec.

[7] Consolaro A, Trevizan M, Consolaro RB, Oliveira IA (2017). Mecanismos de formação da face o que ocorre é nivelamento, e não fusão, dos processos. Rev Clín Ortod Dental Press.

[8] Bishara SE (2000). Textbook of Orthodontics.

[9] Bishara SE (2004). Ortodontia.

[10] Bjork A, Skieller V (1984). Growth and development of the maxillary complex. Inf Orthod Kieferorthop.

[11] Suzuki HM, Previdente LH (2016). Suzuki SS, Garcez AS, Consolaro A Expansão Rápida da Maxila Assistida com Mini-implantes / MARPE: em busca de um movimento ortopédico puro. Rev Clin Ortod Dental Press.

[12] Barteczko K, Jacob M (2004). A re-evaluation of the premaxillary bone in humans. Anat Embryol (Berl).

[13] Woo JK (1949). Ossification and growth of the human maxilla, premaxilla and palate bone. Anat Rec.

[14] Revelo B, Fishman LS (1994). Maturational evaluation of ossification of the midpalatal suture. Am J Orthod Dentofacial Orthop.

[15] Lisson JA, Kjaer I (1997). Location of alveolar clefts relative to the incisive fissure. Cleft Palate Craniofac J.

[16] Noback CR, Moss ML (1953). The topology of the human premaxillary bone. Am J Phys Anthropol.

[17] Hafkamp HC, Bruintjes TD, Huizing EH (1999). Functional anatomy of the premaxillary area. Rhinology.

[18] Trevizan M, Consolaro A (2017). Premaxilla: an independent bone that can base therapeutics for middle third growth. Dental Press J Orthod.

[19] Delaire J (1965). Lateral limits of the os incisivum. Fortschr Kieferorthop.

[20] Baxter DJ, Shroff MM (2011). Developmental maxillofacial anomalies. Semin Ultrasound CT MR.

[21] Precious DS (2009). A new reliable method for alveolar bone grafting at about 6 years of age. J Oral Maxillofac Surg.

[22] Hallman M, Thor A (2008). Bone substitutes and growth factors as an alternative/complement to autogenous bone for grafting in implant dentistry. Periodontol 2000.

[23] Haskell BS, Farman AG (1985). Exploitation of the residual premaxillary-maxillary suture site in maxillary protraction An hypothesis. Angle Orthod.

[24] Vardimon AD, Graber TM, Voss LR, Verrusio E (1987). Magnetic versus mechanical expansion with different force thresholds and points of force application. Am J Orthod Dentofacial Orthop.

[25] Witzig JW, Spahl TJ (1987). The clinical management of basic maxillofacial orthopedic appliances.

[26] Consolaro A, Consolaro MFM-O (2007). Protocolo semanal repetitivo de expansão rápida da maxila e constrição alternadas e técnica da protração maxilar ortopédica efetiva Porque?. Como?. Rev Clín Ortod Dental Press.

[27] Silva E, Meloti F, Pinho S, Gasque CA (2017). Correção da Classe III esquelética em pacientes jovens - Ertty Gap III(r) Orthod Sci. Pract.

[28] Faust RA, Phillips CD (2001). Assessment of congenital bony nasal obstruction by 3-dimensional CT volume rendering. Int J Pediatr Otorhinolaryngol.

[29] Brown OE, Myer CM, Manning SC (1989). Congenital nasal pyriform aperture stenosis. Laryngoscope.

[30] Tagliarini JV, Nakajima V, Castilho EC (2005). Congenital nasal pyriform aperture stenosis. Braz J Otorhinolaryngol.

[31] Rai V, Saha S, Yadav G, Tripathi AM, Grover K (2014). Dental and skeletal maturity - a biological indicator of chronologic age. J Clin Diagn Res.

[32] Carmody KA, Mooney MP, Cooper GM, Bonar CJ, Siegel MI, Dumont ER (2008). Relationship of premaxillary bone and its sutures to deciduous dentition in nonhuman primates. Cleft Palate Craniofac J.

[33] Ten Cate AR (2001). Histologia Bucal : Desenvolvimento Estrutura e Funcao.

[34] Kraus BS, Decker JD (1960). The prenatal inter-relationships of the maxilla and premaxilla in the facial development of man. Acta Anat.

[35] Kvinnsland S (1969). Observations on the early ossification of the upper jaw. Acta Odontol Scand.

[36] Mooney MP, Siegel MI (1986). Developmental relationship between premaxillary-maxillary suture patency and anterior nasal spine morphology. Cleft Palate J.

[37] Mooney MP, Siegel MI, Kimes KR, Todhunter J (1991). Premaxillary development in normal and cleft lip and palate human fetuses using three-dimensional computer reconstruction. Cleft Palate J.

[38] Wood NK, Wragg LE, Stuteville OH (1967). The premaxilla embryological evidence that it does not exist in man. Anat Rec.

[39] Wood NK, Wragg LE, Stuteville OH, Oglesby RJ (1969). Osteogenesis of the human upper jaw proof of the non-existence of a separate premaxillary centre. Arch Oral Biol.

[40] Vacher C, Onolfo JP, Lezy JP, Copin H (2001). The growth of the maxilla in humans. What place for the premaxilla?. Rev Stomatol Chir Maxillofac.

[41] Jacobson A (1957). Embryological Evidence for the Non-Existence of the Premaxilla in Man. Angle Orthod.

[42] Wood-Jones F (1938). The Fate of the Human Premaxilla. J Anat.

[43] Behrents RG, Harris EF (1991). The premaxillary-maxillary suture and orthodontic mechanotherapy. Am J Orthod Dentofacial Orthop.

[44] Macklin C (1921). The skull of a human fetus of forty-three mm greatest length. Contribution to Embriology.

[45] Sejrsen B, Kjaer I, Jakobsen J (1993). The human incisal suture and premaxillary area studied on archaeologic material. Acta Odontol Scand.

[46] Consolaro A (2018). A pré-maxila e a evolução do homem!. Jornal da Cidade.

[47] Botti S, Rumeau C, Gallet P, Jankowski R (2017). Vomero-premaxillary joint a marker of evolution of the species. Eur Ann Otorhinolaryngol Head Neck Dis.

[48] Chen CP, Huang JP, Chen YY, Chern SR, Wu PS, Su JW (2013). Chromosome 18p deletion syndrome presenting holoprosencephaly and premaxillary agenesis: prenatal diagnosis and aCGH characterization using uncultured amniocytes. Gene.

[49] Savastano CP, El-Jaick KB, Costa-Lima MA, Abath CM, Bianca S, Cavalcanti DP (2014). Molecular analysis of holoprosencephaly in South America. Genet Mol Biol.

[50] Maureille B, Bar D (1999). The premaxilla in Neandertal and early modern children ontogeny and morphology. J Hum Evol.

[51] Pryor JW (1928). Difference in the Ossification of the Male and Female Skeleton. J Anat.

[52] Francis CC (1940). Factors influencing appearance of centers of ossification during early childhood II. A comparative study of degree of epiphysial ossification in infancy under varying conditions of diet and health. Am J Dis Child.

[53] Christie AU, Dunham EC, Jenss RM, Dippel A (1941). Development of the center for the cuboid bone in newborn infants a roentgenographic study. Am J Dis Child.

[54] Consolaro A, Consolaro MFM-O (2008). Expansão Rápida da Maxila e Constrição Alternadas (ERMC-Alt) e técnica de Protração Maxilar Ortopédica Efetiva extrapolação de conhecimentos prévios para fundamentação biológica. Rev Dental Press Ortod Ortop Facial.

[55] Spahl TJ (1991). More on premaxillary-maxillary suture. Am J Orthod Dentofacial Orthop.

[56] Schwartz JH (1982). Dentofacial growth and development in Homo sapiens evidence from perinatal individuals from Punic Carthage. Anatomischer Anzeiger.

[57] Wertz RA (1970). Skeletal and dental changes accompanying rapid midpalatal suture opening. Am J Orthod.

[58] Wertz R, Dreskin M (1977). Midpalatal suture opening a normative study. Am J Orthod.

[59] Kadanoff D, Mutafov S, Jordanov J (1969). Anthropological and anatomical characteristics of the bony palate. Gegenbaurs Morphol Jahrb.

[60] Chase W (1942). The early development of the human premaxilla. Am Dent Assoc.

[61] Piersol GA (1930). Human Anatomy.

[62] Scaeffer JP (1942). Morris' Human Anatomy: a complete systematic treatise.

[63] Ashley-Montagu MF (1935). The Premaxilla in the Primates. Quarterly Rev Biol.

[64] Brenner DJ, Doll R, Goodhead DT, Hall EJ, Land CE, Little JB (2003). Cancer risks attributable to low doses of ionizing radiation assessing what we really know. Proc Natl Acad Sci U S A.

[65] Ennes JP (2001). Consolaro A, Ortis MFM, Velloso TRG O periósteo e a ortopedia dos maxilares. Rev Dental Press Ortod Ortop Facial.

[66] Consolaro A (2005). Reabsorções dentárias nas especialidades clínicas.

